# Diffusion‐based size determination of solute particles: a method adapted for postsynaptic proteins

**DOI:** 10.1002/2211-5463.70111

**Published:** 2025-09-01

**Authors:** András László Szabó, Eszter Nagy‐Kanta, Soma Varga, Edit Andrea Jáger, Csaba István Pongor, Mária Laki, András József Laki, Zoltán Gáspári

**Affiliations:** ^1^ Faculty of Information Technology and Bionics Pázmány Péter Catholic University Budapest Hungary; ^2^ Department of Public Health Sciences, Faculty of Health Sciences Semmelweis University Budapest Hungary

**Keywords:** bioinformatics, fluorescent microscopy, microfluidics, postsynaptic density

## Abstract

The postsynaptic density (PSD) is a complex, multilayered protein network largely situated on the internal surface of the postsynaptic membrane. It is the first processing unit for incoming synaptic signals, and changes in its internal structure are associated with synaptic strength and plasticity. These structural changes are largely governed by multivalent interactions between its components. The *in vitro* characterization of such complexes requires unbiased methods that can be used to estimate the size of the emerging assemblies for systems with multiple possible stoichiometries. Here, we present an experimental method for detecting specific PSD proteins as well as their complexes based on their diffusion in a microfluidic environment. The method requires a fluorescent labeling technique that does not disrupt the function of labeled proteins, a microfluidic device that can maintain laminar flow for protein solutions, a microscope that can record the fluorescent signal emitted by these solutions, and an analytic software package that can process the collected experimental data and convert them into approximate particle sizes. We demonstrate the applicability of our method on protein constructs of various postsynaptic proteins, including the multivalent assembly between GKAP and LC8.

## Graphical abstract

We present a diffusion‐based approach for measuring the size of macromolecules and their complexes, and demonstrate its use on postsynaptic proteins. The method requires fluorescein‐labelled protein samples, a microfluidic device that maintains laminar flow for said samples, a microscope recording the emitted fluorescent signals, and an analytic software package that converts the measured data into approximate particle sizes.

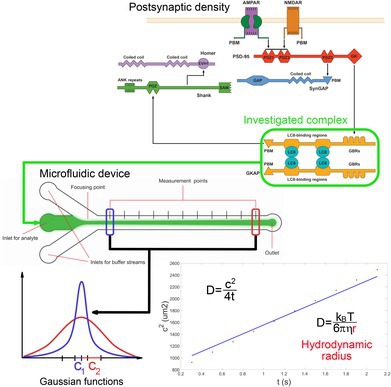

**Abbreviations**
AMPAR: α‐amino‐3‐hydroxy‐5‐methyl‐4‐isoxazolepropionic acid receptor
BSA: bovine serum albumin
D233: human Drebrin construct, residues 233‐317
DLS: dynamic light scattering
EGFP: enhanced green fluorescent protein
FITC: green‐fluorescent fluorescein‐EX
GKAP: guanylate kinase‐associated protein
GKAP‐DLC2: GKAP's two LC8(dynein light chain)‐binding motifs
GKAP‐PBM: GKAP's PDZ‐binding motif
GoF: goodness of fit metric
IDP: intrinsically disordered protein
IEC: ion exchange chromatography
LC8: dynein light chain LC8 protein
LLPS: liquid–liquid phase separation
MAGUK: membrane‐associated guanylate kinase
MLO: membraneless organelle
NMDAR: N‐methyl D‐aspartate receptor
PDMS: polydimethylsiloxane
PSD: postsynaptic density
PSD‐95: postsynaptic density protein 95
RMSE: root mean squared error
SEC‐SAXS/MALLS: size exclusion chromatography – small angle X‐ray scattering / multi‐angle laser light scattering
SSE: squares due to error
SUV: small unilamellar vesicle
SynGAP: synaptic Ras GTPase‐activating protein 1
TEV: tobacco etch virus

## Organization of the postsynaptic density

The postsynaptic density (PSD) is a multilayered cellular component situated on the internal surface of postsynaptic membranes. These disk‐shaped compartments are rich in proteins and nucleic acids, including RNA‐binding proteins, actin filaments, membrane‐associated guanylate kinases (MAGUKs), and scaffolds [1, 2]. Their primary constituents are the guanylate kinase‐associated protein (GKAP), the postsynaptic density protein 95 (PSD‐95), various Shank and Homer proteins, and the synaptic Ras GTPase‐activating protein 1 (SynGAP) [3, 4, 5, 6]. PSDs are also associated with transmembrane proteins, such as the N‐methyl D‐aspartate receptor (NMDAR) [7]. They are the primary cellular elements that process incoming synaptic signals, and their structural changes exhibit a strong correlation to synaptic strength and plasticity, which are pillars of higher biological functions such as memory and learning [8]. The functional importance of PSDs is further emphasized by studies of the wake/sleep cycle suggesting that synaptic strength is renormalized during sleep [9].

The structural organization of PSDs is governed by various complex biochemical processes, including liquid–liquid phase separation (LLPS). Protein phase separation is a complex biochemical process, typically initiated by multivalent interactions between multidomain proteins and RNAs. The phenomenon produces the so‐called membraneless organelles (MLOs) that often play the role of dynamically changing storage units for the components of certain cellular processes, such as RNA transcription, chromatin regulation, or the organization of PSDs [8, 10, 11]. LLPS is a specific type of protein phase separation characterized by the formation of distinct phases where certain solutes appear in high concentrations, resulting in liquid‐like properties. This particular type of the phenomenon is driven by multivalent proteins appropriately referred to as “drivers.” [12] The role of LLPS in structural organization is indicated by an experimental model constructed from two abundant components of PSDs, SynGAP, and PSD‐95. This simple model has been observed to self‐organize into highly condensed, PSD‐like droplets *in vitro* [13]. The importance of phase separation in the organization of PSDs has since been reinforced by more complex *in vitro* models, one including GKAP, Shank3, and Homer3, in addition to SynGAP and PSD‐95 [14]. Structural investigation of the higher‐order complexes of the postsynaptic density, formed by multiple polypeptide chains, is necessary for the detailed understanding of the molecular events behind learning, memory, and cognitive dysfunctions.

## Biological significance of the methodology presented in this work

**Fig. 1:**
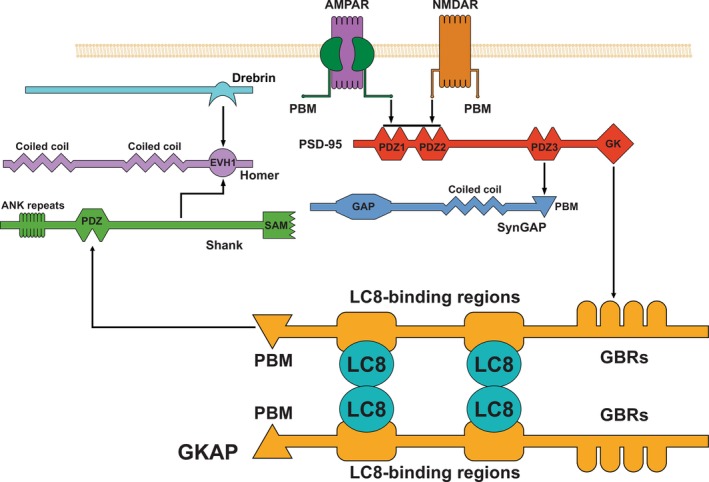
Schematic illustration for an *in vitro* postsynaptic density model, consisting of AMPARs (α‐amino‐3‐hydroxy‐5‐methyl‐4‐isoxazolepropionic acid receptors), NMDARs (N‐methyl D‐aspartate receptors), the postsynaptic density protein 95 (PSD‐95), the synaptic Ras GTPase‐activating protein 1 (SynGAP), the guanylate kinase‐associated protein (GKAP), the dynein light chain LC8 protein (LC8), the protein Drebrin, as well as members of the Homer and Shank protein families. Some of these proteins have PDZ domains, PDZ‐binding motifs (PBMs), guanylate kinase domains (GKs), Ras‐GAP domains (GAPs), guanylate kinase‐binding regions (GBRs), ankyrin (ANK) repeats, sterile alpha motifs (SAMs), and EVH1 domains. Proteins are depicted in arbitrary orientations, serving a simpler representation of the selected interactions. This study primarily focuses on GKAP's interactions within this network, specifically its PDZ‐binding motif and the hexameric complex formed with LC8 dimers that bind to GKAP molecules at multiple positions.

It is clear that, in theory, a number of different assemblies can be formed between different partners within the elaborate network of the PSD, and the exact stoichiometry and thus the size of these assemblies might be variable. Investigation of such complexes, their heterogeneity, and the regulation of the potential rearrangements in the higher order complexes, for example, upon posttranslational modifications, requires sensitive methods capable of distinguishing between assemblies of different sizes. Such a method can be based on the size‐dependent diffusion properties of the protein and their complexes. To explore these aspects, the diffusion‐based analytic method presented in this work was tested on a two‐component subsystem of the simplified postsynaptic protein network depicted in Fig. 1. One of the components is GKAP, a known scaffolding protein that participates in the regulation of NMDA receptors. It contains a high ratio of disordered regions and multiple binding sites, two of which had been identified as LC8 binding motifs [15]. The other component is the dynein light chain LC8 protein that can form multivalent interactions with various intrinsically disordered regions and proteins (IDRs/IDPs) [16]. It is also known to form dimers that can bind two additional ligands, suggesting potential for the initiation of MLO‐formation through multivalent interactions. This potential is further supported by the level of disorder within GKAP and the binding partners of LC8, since IDPs are generally prone toward phase separation, as observed in the case of the RNA‐binding protein Fused in sarcoma (FUS), the TAR DNA‐binding protein 43 (TDP‐43), and the intrinsically disordered intracellular domain of Nephrin (NICD) [17, 18, 19]. GKAP and LC8 are also known to form hetero‐oligomeric complexes, the exact stoichiometry of which has only been explored recently [20, 21]. Based on our understanding of GKAP‐LC8 complexes, the two‐component system constructed for testing the diffusion‐based analytic method consisted of LC8 and GKAP molecules with a 4 : 2 stoichiometry (2 : 2 stoichiometry of LC8 dimers and GKAP monomers). The GKAP construct containing both LC8‐binding regions is referred to as GKAP‐DLC2. GKAP's PDZ‐binding motif (its construct referred to as GKAP‐PBM) was also investigated, as a stand‐alone particle at this point. Apart from GKAP and LC8, the protein Drebrin has also been analyzed via this method, though only as a stand‐alone particle, as it does not exhibit direct interactions with GKAP or LC8, but with the EVH1 domain of Homer, another component of the PSD. The Drebrin construct used in this study is referred to as D233 [22].

## Challenges and current approaches for the investigation of dynamic protein complexes

Experimental characterization of protein–protein interactions often relies on the characterization of simplified systems. With the exception of relatively small globular proteins, in many cases, only specific segments of one or both partners are used. However, as detailed above on the example of the postsynaptic density, a large portion of human proteins contain long disordered segments, often with multiple binding sites, and the exact size, shape, and composition of physiologically occurring complexes is determined by the interplay between the individual binding events to these [23]. In multivalent scenarios, binding between the same partners might occur at different sites, and there might be a dynamic interchange between the individual interactions, in extreme cases leading to the emergence of so‐called fuzzy complexes [24]. The stoichiometry of the emergent multivalent complexes might also be variable, depending on the presence of additional structural constraints. Detailed characterization of dynamic complexes is possible using NMR spectroscopy, which provides information at the atomic level on both the structure and the dynamics of the complexes, but also requires isotope‐labeled samples [25]. To provide independent observations, additional methods are needed that can estimate the size(s) of the complexes formed in a reliable manner. However, most of the currently applied methods, like SEC‐SAXS/MALLS (size exclusion chromatography—small angle X‐ray scattering/multi‐angle laser light scattering) and DLS (dynamic light scattering) work well for homogeneous samples [26, 27]. For samples with heterogeneous composition, previous developments have demonstrated the potential of microfluidics‐based approaches. In the method published by Arosio *et al*. [28], green fluorescent polystyrene particles, α‐synuclein molecules, antibody fragments, and other nanoparticles were injected into a microfluidic device in order to measure their lateral movement via diffusion. Based on these measurements, the hydrodynamic radii of the particles could be approximated and compared to *a priori* data generated with DLS. This technique was also explored by Gang *et al*. [29] with polydisperse mixtures including up to three components with distinct sizes: α‐synuclein fibrils, small unilamellar vesicles (SUVs), and SUVs with α‐synuclein fibrils bound to the external surface of their membranes. However, these approaches require *a priori* knowledge of the particle sizes present in the system, limiting their applicability to characterize previously unexplored assemblies and their use to provide independent estimates supporting other investigations.

## Overview and applicability of the developed experimental approach

Experimental characterization of dynamic protein interactions and previously uncharacterized complexes that might give rise to heterogeneous molecular populations requires methods that provide an estimate of the complex size(s) without *a priori* hypotheses, work well in aqueous solutions, and require no or only subtle modification of the investigated molecules. Inspired by the microfluidics‐based techniques described previously, we present a method that meets these criteria. A key aspect of our approach is that it does not require an *a priori* hypothesis on the size(s) of the particle(s) investigated besides the expected, relatively wide range, as it uses a first‐principles diffusion model to which the experimental data can be fit. As for the device, we settled on a microfluidic focuser extended with a long straight channel. In contrast to longer channels used in previous approaches, the elimination of curvatures in our layout can arrange the investigated particles into a predetermined position and maintain their laminar flow thereafter, which is crucial to measuring their lateral diffusion (see Theory behind the applied diffusion analysis in the Methods section). The only required sample modification is labeling one of the protein components in the complex with fluorescein. This labeling technique provides sufficient fluorescent signals for the proteins and their complexes to be monitored without disrupting the underlying interactions. Our solution provides a robust, hypothesis‐free method to estimate the sizes of proteins and their complexes with a precision higher than that of DLS (see Size approximation in the Results section).

## Methods

### Theory behind the applied diffusion analysis

In solutions, unrestricted particles are in perpetual motion via diffusion. The smaller they are, the faster they move, as described by the Stokes–Einstein equation:

(1) $$D=\frac{k_{B}T}{6\pi \mathrm{\eta r}}$$

where *D* is the diffusion coefficient of the particle, *k*_B_ is the Boltzmann constant, *T* is the absolute temperature, *η* is the viscosity of the (liquid) medium, and *r* is the hydrodynamic radius of the (spherical) particle. It is important to note that this equation only works in the case of globular particles and a liquid with low Reynolds number, which is characteristic of laminar flow. Therefore, the method requires a device that achieves laminar flow for protein samples, from now on referred to as analytes. Additionally, the device has to arrange particles into a predetermined position from where they can freely diffuse during the laminar flow. A microfluidic focuser with the appropriate layout can achieve these criteria by compressing the analyte into the middle of a channel with buffer streams from both sides.

This method is also based on the assumption that diffusing particles would approximate normal distribution. Since their motion is primarily governed by diffusion in directions that are perpendicular to the laminar flow's direction, it is logical to examine it in one such direction. Thus, the distribution of particles can be described through Brownian motion with one spatial dimension:

(2) $$\rho \left(x,t\right)=\frac{N}{\sqrt{4\pi \mathrm{Dt}}}\;e^{-\frac{x^{2}}{4\mathrm{Dt}}}$$

where *t* is the time elapsed since diffusion began, *N* is the number of particles that start from the origin at time *t* = 0, *x* is the distance from the origin, and *ρ* is the particle density at distance *x* at time *t*. Concurrently, the mathematical description of a Gaussian function is as follows:

(3) $$f\left(x\right)=ae^{-{\left(\frac{x-b}{c}\right)}^{2}}$$

where *a* is the amplitude, *b* is the center, and c is the standard deviation of the bell curve, while *f*(*x*) is the value of the function at position *x*. If the time component in Eq. 2 is set to a constant value, then Eqs 2 and 3. match as

(4) $$\frac{N}{\sqrt{4\pi \mathrm{Dt}}}\;e^{-\frac{x^{2}}{4\mathrm{Dt}}}=ae^{-\frac{x^{2}}{c^{2}}}$$

If the Gaussian function is centered on the origin (*b* = 0), the following expressions can be derived from Eq. 4:

(5) $$c^{2}=4\mathrm{Dt}$$

(6) $$a=\frac{N}{\sqrt{4\pi \mathrm{Dt}}}$$

From Eqs 5 and 6. the diffusion coefficient *D* and the number of particles *N* can be written as follows:

(7) $$D=\frac{c^{2}}{4t}$$

(8) $$N=\mathrm{ac}\sqrt{\pi }$$

**Fig. 2:**
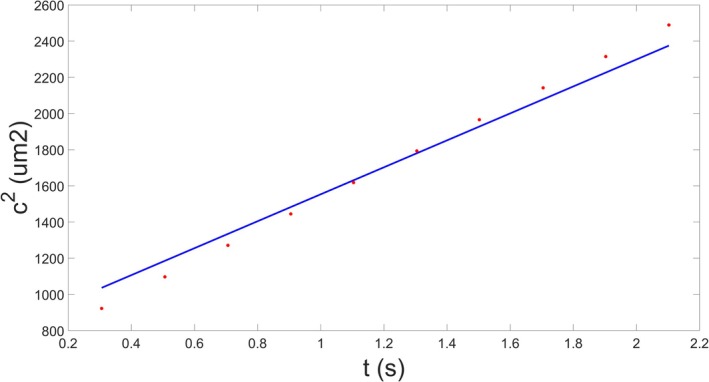
The variance of Gaussian functions (*c* ^2^) fitted to fluorescent intensity profiles measured at different distances from the front of the channel (where the analyte was focused). The *x*‐axis shows the average time (*t*) it takes for the particles to reach a given measurement point at a constant flow rate (see Measurement protocol). The analyte was the enhanced green fluorescent protein; the figure represents a single biologically independent replicate (*n* = 1). The resulting points are fitted with a linear function, the incline of which is directly proportional to the diffusion coefficient of solute particles in the analyte.

And so, the number of particles can be approximated if the amplitude and standard deviation of the Gaussian function describing their distribution at a given point are known. More importantly, their diffusion coefficient, and thus their size, can be approximated as the incline of the linear function fitted to the points given by the Gaussian functions' variances and the associated time components (Fig. 2). This requires multiple measurements at multiple points in time during the laminar flow, which can be achieved by setting up multiple measurement points along a straight channel added to the microfluidic focuser.

### Device fabrication

The molds for microfluidic devices were fabricated using soft lithography and polydimethylsiloxane (PDMS) replica molding techniques. In this process, a negative photoresist height of 20 μm (SU‐82015; Microchem Corp., Newton MA, USA) is applied to the top of a silicon wafer by spin‐coating (WS‐650‐23; Laurell Technologies Corp., Lansdale PA, USA). Channel design was applied to the surface by laser writing (μPG 101, Heidelberg Instruments Mikrotechnik GmbH, Heidelberg, Germany). After the development of the mold, the PDMS (SYLGARD 184 Silicone Elastomer Kit; Dow Corning, Midland, MI, USA) base and curing agent were mixed in a 10 : 1 ratio, degassed, poured over the mold, and cured at 70 °C for 90 min. Once the polymerization process is complete, the PDMS was removed from the mold surface, and the inlets and outlets were processed. The PDMS slice containing the microfluidic channel was bonded to a glass slide using plasma treatment (Zepto, Diener electronic GmbH, Ebhausen, Germany).

PTFE tubing (Masterflex Microbore Transfer Tubing MFLX06417‐11, Cole‐Parmer Instrument Company, LLC, Vernon Hills IL, USA) was inserted into the device inlets and outlets, then connected to 2‐mL syringes and NE‐1002X syringe pumps via a 27 Gauge needle tip (27 Gauge Clear Precision Tip 0.5″ Long; Adhesive Dispensing, Ltd, Milton Keynes, UK). The PTFE tube on the outlet is approx. 15 cm long and leads into a beaker that is also positioned on the stage of the microscope. The PTFE tubes on the inlets are approx. 25 cm in length, which is the minimum length required to keep the tubing from becoming taut as the stage moves the device around, minimizing the risk of it slipping out of the inlets.

### Protein expression and purification

The GKAP‐DLC2 construct was designed to include both LC8‐binding motifs of GKAP with extended flanking regions (10 residues on the N terminus, 14 residues on the C‐terminus). The segment spanning residues 655–711 in the *Rattus norvegicus* GKAP isoform 3 “GKAP1a” (UniProt ID: P97836‐5) was selected. The GKAP‐PBM construct incorporated the PDZ‐binding motif EAQTRL and its extensive flanking region of 37 additional residues. Both inserts were cloned to an altered pEV vector (Novagen, Madison, WI, USA) that contains an N‐terminal 6×His tag and a tobacco etch virus (TEV) protease cleavage site. The actual constructs contained four extra residues (GSHM) at the N terminus, remaining from the expression tag. The *Rattus norvegicus* DYNLL2 gene (UniProt ID: Q78P75) in the pEV plasmid vector is 100% identical to the human ortholog (UniProt ID: Q96FJ2). The pEV vector also contains an N‐terminal 6xHis tag, the TEV protease cleavage site, and four residues (GSHM) at the N terminus.

All three protein constructs were produced in BL21 (DE3) *E. coli* (Novagen) cells, transformed with the vectors, grown in LB media, induced with 1 mm IPTG (Isopropyl β‐D‐1‐thiogalactopyranoside) at 6 MFU cell density, and the recombinant proteins were expressed at 20 °C overnight. The centrifuged cell pellets were stored at −20 °C for further usage. Cell pellets were lysed by ultrasonic homogenization in 10% cell suspension using a lysis buffer (50 mm NaPi, 300 mm NaCl, pH 7.4). In the case of GKAP‐DLC2, denaturing–renaturing IMAC purification was applied denatured with 6 m GdnHCl, 50 mm NaPi, added to 5 mL Nuvia™ Ni‐affinity column (Bio‐Rad Laboratories Inc., Hercules, CA, USA), then bound proteins were renatured with native buffer (50 mm NaPi, 20 mm NaCl, pH 7.4). After washing, elution was performed with 250 mm imidazole and was followed by His‐tag removal with TEV protease. For LC8 and GKAP‐PBM, the same purification protocol was used, but after ultrasonic homogenization and centrifugation, the supernatant was immediately purified with IMAC Nuvia Ni‐affinity column without denaturation–renaturation.

Protein samples were concentrated by ultrafiltration using Amicon® Ultra Centrifugal Filter with 3 kDa molecular weight cutoff value, and the buffer was changed to low salt NaPi Buffer (50 mm NaPi, 20 mm NaCl, pH 6.0). Proteins, except GKAP‐PBM, were further purified by ion exchange chromatography (IEC), using 5 mL High Q column with the same buffer (50 mm NaPi, 20 mm NaCl, pH 6.0). Recombinant proteins were collected in the flow through fraction. After another step of protein concentration, proteins were further purified with size exclusion chromatography (SEC) on a Superdex™ 75 Increase 10/300 GL 24 mL column, the buffer was 50 mm NaPi, 20 mm NaCl, pH 6.0. Later 5 mm TCEP (pH adjusted to 7.4 with NaOH) was added to GKAP‐DLC2 and LC8 samples individually. The concentration of LC8 and GKAP‐PBM was measured by its absorbance at 280 nm using a NanoDrop2000 photometer, while the concentration of GKAP‐DLC2 + LC8 hexamers was measured with Qubit Protein assay. The molecular weight of GKAP‐DLC2, LC8, and GKAP‐PBM monomers was determined to be 7.01 kDa, 10.6 kDa, and 5.2 kDa, respectively, validated via SDS/PAGE.

Drebrin (D233) was produced by using the full sequence in the *Homo sapiens* isoform Q16643 as a template for cloning the segment 233 to 317 into NdeI and HindIII sites of a modified pET‐15b vector along with an N‐terminal 6xHis‐tag and a TEV cleavage site (ENLYFQG). This construct was also produced in BL21 (DE3) *E. coli* cells and grown in LB media. After inducing with 1 mm IPTG, cells were incubated for 3 h at 37 °C before harvesting by centrifugation. The lysis buffer (50 mm NaPi, 300 mm NaCl, 5 mm β‐mercaptoethanol, pH 7.4) also contained 1 mm AEBSF protease inhibitor cocktail (AEBSF Protease Inhibitor #78431; Thermo Fisher Scientific Inc., Waltham, MA, USA). The same buffer, without the protease inhibitor cocktail, was added to the Ni‐affinity column while applying IMAC purification to D233. Elution was performed with 500 mm imidazole, and the His‐tag was cleaved with TEV protease. The sample was further purified via SEC on a Superdex™ 75 Increase 10/300 GL 24 mL column equilibrated with buffer suitable for FITC labeling (50 mm NaPi, 20 mm NaCl, pH 8.0). The molecular weight of D233 was determined to be 10.32 kDa, reinforced by SDS/PAGE.

### Preparation of samples

Protein samples were labeled with the Green‐fluorescent Fluorescein‐EX (FITC) labeling kit (ThermoFisher cat. num. F10240) using the following protocol: The buffer of GKAP‐DLC2, GKAP‐PBM, and D233 was changed to 50 mm NaPi, 20 mm NaCl, pH 8.0 as suggested by the manufacturer. Based on absorbance measurement with Nanodrop, the concentration was 3.5 mg·mL^−1^ and 2 mg·mL^−1^, respectively. 0.5 mL and 1.5 mL samples were added to one vial containing the reactive dye, respectively. After 1 h of incubation and stirring at room temperature, the vials were stored at 4 °C overnight (for 16 h). The preparation of Drebrin samples slightly diverged here, only being incubated for 1 h. After the labeling reaction, any unbound reactive dye was separated from the labeled protein with size exclusion chromatography (SEC). 0.5 mL samples were injected one by one to a Superdex™ 75 Increase 10/300 GL 24 mL column; the flow speed was between 0.8 mL·min^−1^ (with the same buffer as used for the labeling: 50 mm NaPi, 20 mm NaCl, pH 8.0). Unlabeled LC8 dimers were added to the fluorescein‐labeled GKAP‐DLC2 with 2:2 stoichiometry (i.e., 2 LC8 dimers to 2 GKAP‐DLC2 monomers). The final volume of labeled protein solutions had to be at least 300 μL per measurement. All protein samples were focused in the microfluidic device with low salt NaPi buffer streams (50 mm NaPi, 20 mm NaCl, pH 8.0).

Additionally, Enhanced Green Fluorescent Protein (EGFP) was used for testing the various iterations of the microfluidic device. EGFP analytes were focused with a PBS buffer (137 mm NaCl, 2.7 mm KCl, 10 mm Na_2_HPO_4_, and 1.8 mm KH_2_PO_4_, pH 7.4). The analytes were prepared by diluting 2 mg·mL^−1^ EGFP stock solutions with an equal amount of PBS buffer. The 1% BSA solution used for the surface treatment of microfluidic devices was prepared in stock by dissolving 0.05 g BSA powder (VWR Chemicals Bovine Serum Albumin, cat. no. 97061‐420) in 50 mL of PBS buffer, then aliquoted to 1‐mL Eppendorf tubes to be frozen for later use.

### Measurement protocol

**Fig. 3:**
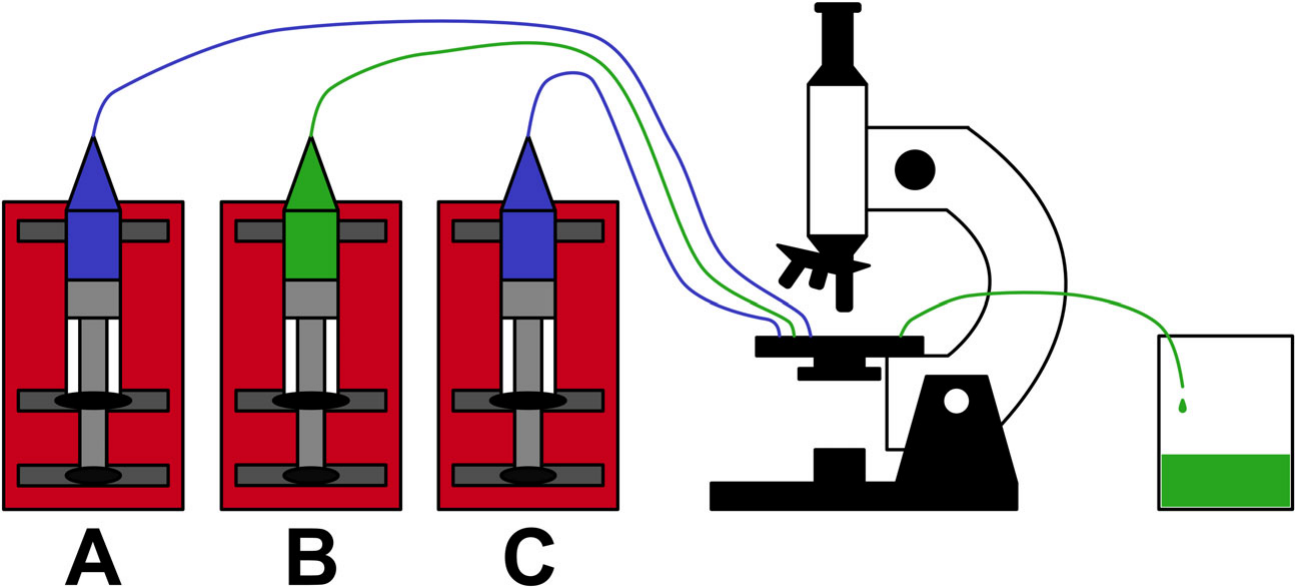
Illustration of the experimental setup that involves three syringe pumps (red), syringes filled with analyte (green) and buffer solutions (blue), a microscope, and a collection cup.

The measurement requires three syringe pumps, connected to a microfluidic device that is placed into a Nikon Ti‐2 E inverted microscope (Fig. 3). The microscope has a motorized stage and filter turret that detects signals passing through a FITC filter (Excitation: 480/30, Dichroic mirror: 505, Barrier filter: 515) with an Andor Zyla 4.2 camera. A Nikon CFI Plan Apochromat Lambda D 20X lens is used because it is not possible to record the entire 300 μm wide main channel at higher magnifications.

**Fig. 4:**
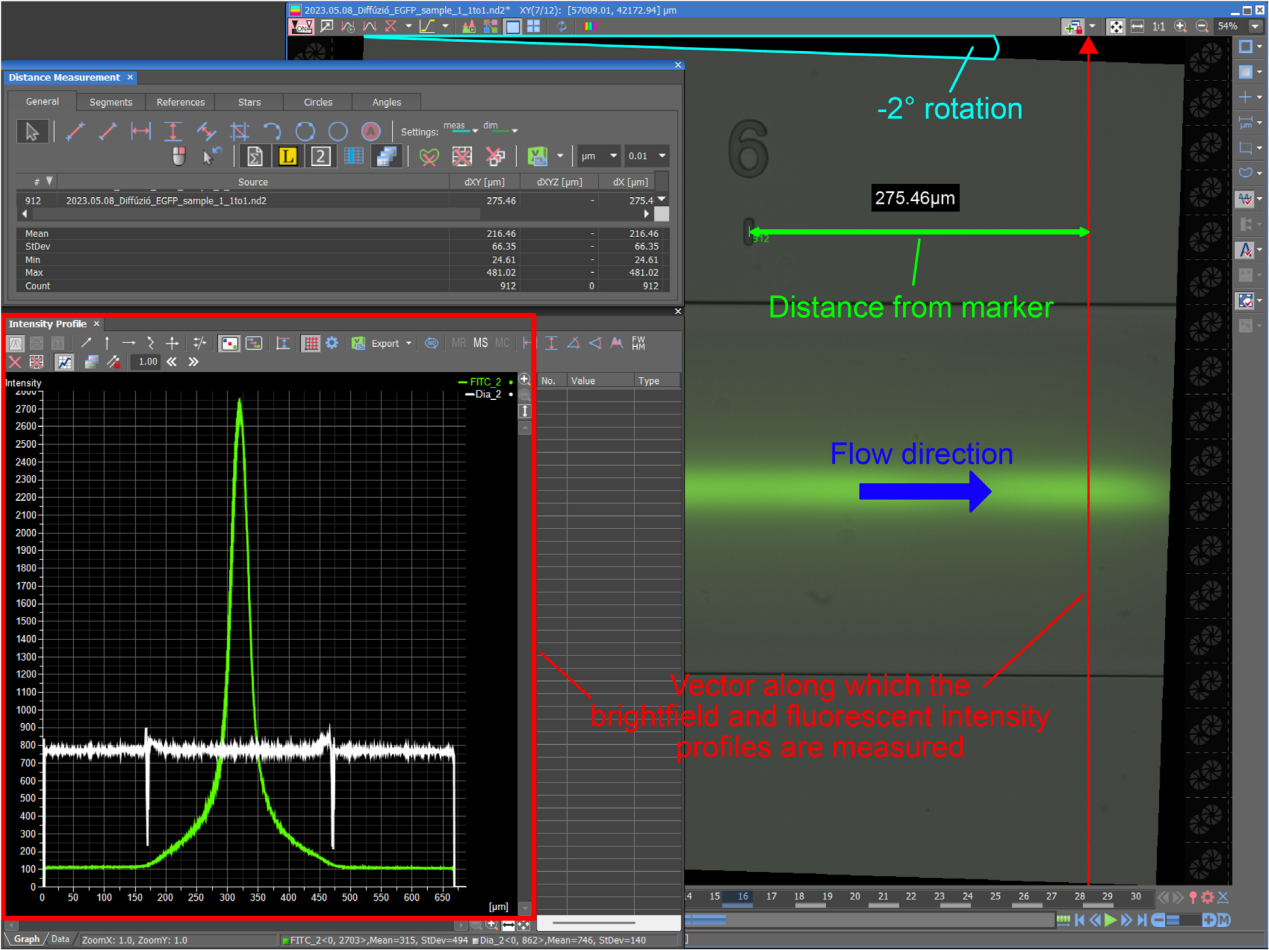
The combined image of a brightfield and fluorescent recording that has been rotated by –2 ° (cyan) so that the vector along which the intensity profiles are measured (red) is perpendicular to the flow direction (blue). The exact position of the vector is recorded in relation to the marker denoting the measurement point (green).

Degassing the system with BSA takes 15 min after all three solutions have reached the device. The following BSA treatment takes an additional 10–15 min. The replacement of syringes in pump A and C is followed by another round of degassing (15 min). The bright‐field images are recorded at this time. The flow is slowed down again (10–15 min) before the BSA in pump B is replaced by the fluorescent analyte. External light sources are minimized around the microscope, and the intersection of the device is monitored at an exposition rate of 300 ms and with 4× gain. The moment the analyte reaches the intersection, its flow rate is reduced. After the system has reached its steady state (10–15 min), the fluorescent images are recorded at an exposition rate of 1–5 s (depending on signal strength) and with 1× gain. The recorded images are converted into intensity profiles in the microscope's software (Fig. 4). The metadata of the measurement is saved in the first tab of an Excel file, while each additional tab contains the intensity profiles of a different measurement point in order from the intersection toward the outlet.

### The analytic software package

The measured experimental data are processed with a software package consisting of custom scripts and functions written in MATLAB. The main script takes a single Excel file containing experimental data as input. It omits any specified measurement points from the evaluation, for example, the last profile should be omitted due to the laminar flow being disturbed by some sort of contaminant stuck between the last two measurement points. The following information are imported from the input file: the width and height of the main channel (μm), the thickness of its side walls as displayed in the bright‐field recording (μm), the flow rate of the analyte and the buffer solutions (μL·min^−1^), the temperature around the stage (*K*), the viscosity of the analyte (Pa*s), the exposition time (s), the position of each profile, relative to the origin point at the intersection (μm), and the fluorescent and bright‐field intensity profiles (1). The script calculates the average velocity of particles:

$$v_{\text{particles}}=\text{flow}/\left(h*w\right)$$

The average time it took for particles to reach each measurement point after reaching the origin point at the intersection is calculated by dividing the distance of the profile from the origin point by the average velocity above. After that, the average time particles spent between consecutive measurement points is yielded by simple subtractions. Since all particles can be assumed to have a hydrodynamic radius between 1 nm and 2 μm, it is possible to calculate the minimum and maximum incline for the linear function fitted to the time‐variance function (see Basic principles of the experimental method).

**Fig. 5:**
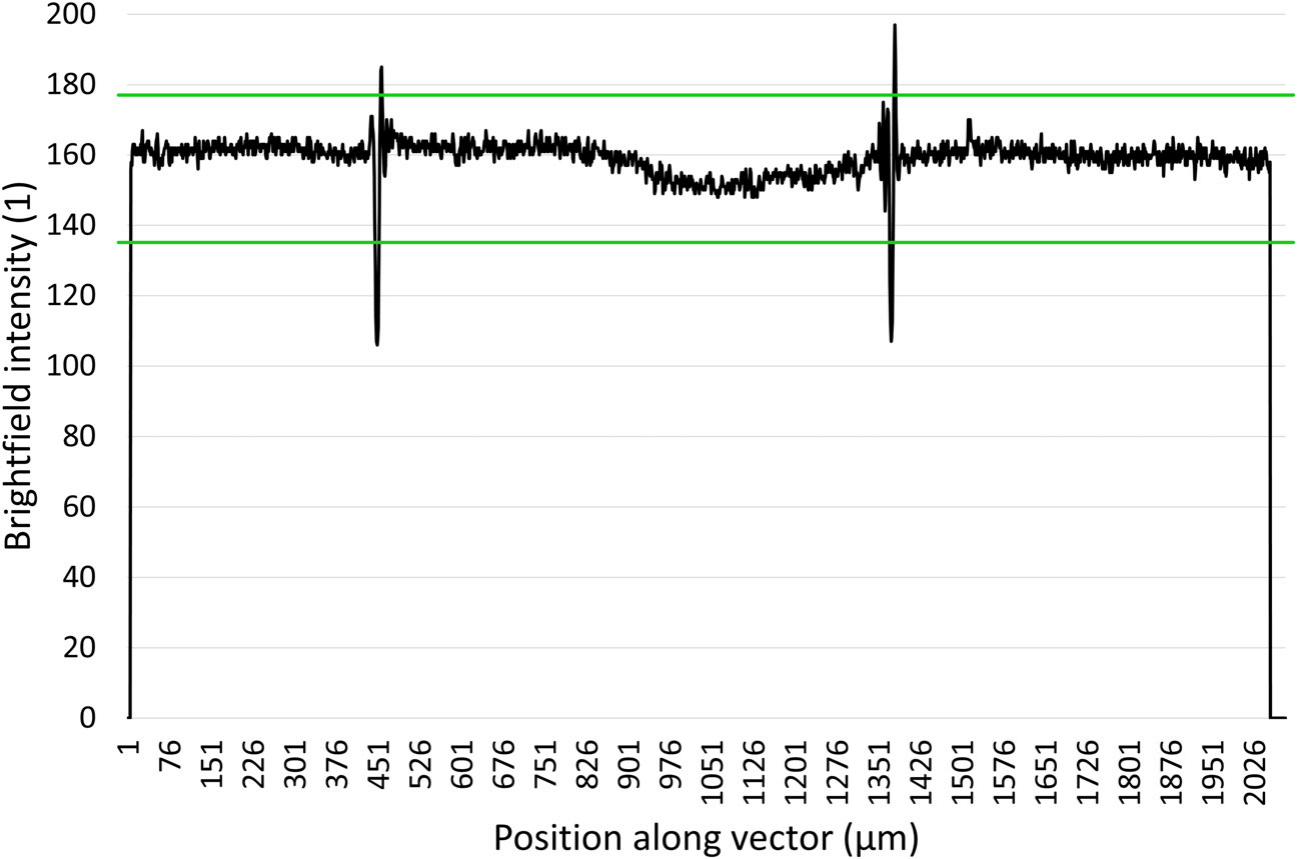
Brightfield intensity profile of a FITC+GKAP‐DLC2 (the guanylate kinase‐associated protein's dynein light chain LC8 protein‐binding motifs, labeled with green‐fluorescent fluorescein‐EX dye) complex sample (black) with horizontal lines (green) at its average minus its standard deviation (135.66) and at its average plus its standard deviation (177.53). The intensity drops to zero toward both ends where the vector along which the intensity is measured goes outside the image. The two large spikes at 376–526 μm and 1276–1426 μm are the sidewalls of the channel that become relevant during the normalization process. The figure shows a single representative measurement (*n* = 1).

In case the recorded images had to be rotated to align them horizontally, the intensity profiles fall to zero at the edges where the vectors along which they were taken include points outside the images. A custom cropping function is called to remove these outside sections. They are recognized by defining a band around the bright‐field profile (Fig. 5). The cropping function starts from the two ends of the bright‐field profiles and removes each point until it comes across one the intensity of which is higher than the lower limit of the band.

**Fig. 6:**
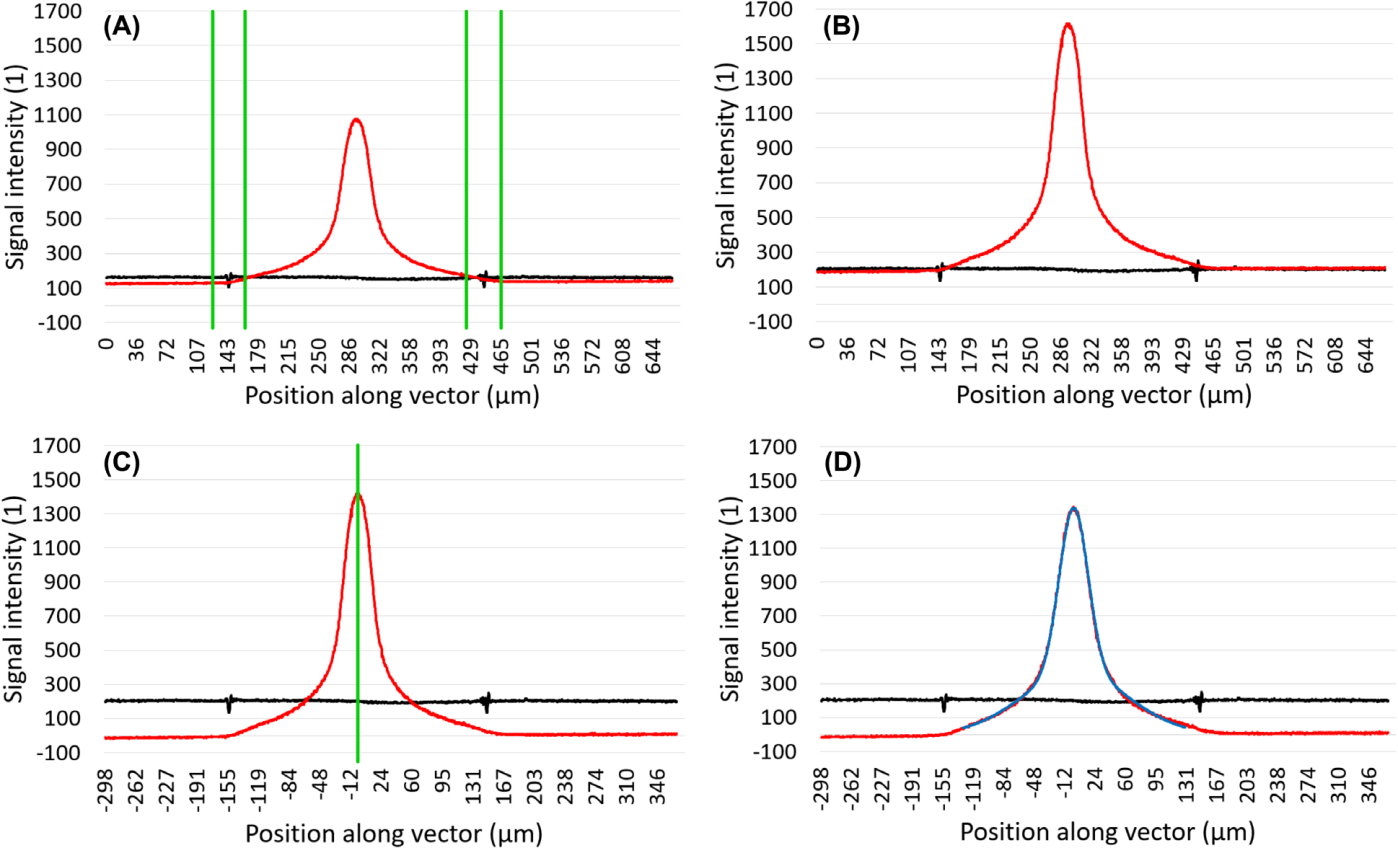
Four major steps from cropping the brightfield (black) and fluorescent (red) profiles to fitting Gaussian functions. The analyte was the guanylate kinase‐associated protein's two dynein light chain LC8 protein‐binding motifs, labeled with green‐fluorescent fluorescein‐EX dye. The figure shows a single representative measurement (*n* = 1). (A) Sections around the spikes in the brightfield profile (bounded by green vertical lines) reveal the positions of the channel's two sidewalls that appear as 20 μm thick lines in the recorded images. The thickness of the sidewalls is doubled to account for the uncertainty in the relation between a spike's position and the middle of the corresponding line. The result is three distinct sections in the fluorescent intensity profile: one measuring the intensity inside the channel and two measuring the intensity outside of it. (B) Both brightfield and fluorescent profiles are multiplied by their respective coefficients to move their baselines (their mean values outside the channel) to the standard value of 200. (C) The fluorescent profile is shifted downwards so that its baseline is aligned with the *x*‐axis (Signal intensity = 0). It is then shifted to the left, so that its peak value (green vertical line) is aligned with the y‐axis (Position = 0). The resulting fluorescent profile is similar to a Gaussian curve that is centered around the *y*‐axis (*x* = 0). (D) A linear combination of two Gaussian functions (blue) is fitted to the fluorescent profile (only the part inside the channel).

After cropping, a custom normalization function is called that brings the signals measured outside the microfluidic channel to the same baseline, and then it aligns that baseline to the (intensity = 0) axis as well as centers the profiles around the fluorescent intensity maximum (Fig. 6). All these steps require the positions of the channel's sidewalls in order to distinguish data points inside it from those outside it. This process starts with finding the position of the fluorescent maximum, from which the function iterates through data points in both directions in the associated bright‐field profile until it comes across either the intensity minimum or maximum in that portion of the profile, whichever is further away from the mean value of the bright‐field intensities. Since the cropping function previously removed data points outside of the recorded images, the only two sections in each bright‐field profile where the intensity significantly deviates from the mean value are the two side walls. Based on the wall thickness defined in the input file, the function excludes sections around these two points, dividing each profile into three sections: the section inside the channel, the right‐hand side external section, and the left‐hand side external section, defined relative to the flow direction. Each bright‐field profile is multiplied by a coefficient to align it with the 200‐intensity line, which is the most common baseline based on observations. The coefficient is simply the ratio of 200 to the mean value of data points inside the channel. In the case of fluorescent profiles, normalization means a two‐step process. The first step is aligning the baseline of each profile to the standard 200, same as before. The goal of this step is to remove the differences between profiles resulting from the slight changes in external noise. The second step is to multiply each profile by a coefficient so that all of them have the same area under their curves. The coefficient in this case is the ratio of the largest integral value to the integral of the current profile. These integrals ignore the sections outside the channel. Finally, the baseline of each fluorescent profile is aligned with the (intensity = 0) axis by subtracting the mean value of outside data points from the entire profile and is centered on the position of its peak value, with a horizontal shift applied to the associated bright‐field profile as well. The position of the peak value is often different from what would be the center of the curve; however, this slight discrepancy is nullified in the next step.

Each profile is then fitted with a Gaussian function or the linear combination of up to eight Gaussian functions, using MATLAB's “prepareCurveData” and “fit” functions (Fig. 6). The parameters of the fitted functions are limited as follows: the amplitude of the curve must not be negative, the center of the curve must be between −5 and 5 μm (the peak value is always within 5 μm of the center of the curve based on observations), and the standard deviation of the curve must be positive but not larger than 100, an upper limit selected because it has been observed to yield the best approximations for samples with known particle sizes. The limits of the standard deviation are adjusted for each profile based on the maximal and minimal changes theoretically possible between measurement points, based on the maximum and minimum incline calculated at the beginning of this section. This ensures that the fitted curves widen as we progress from the first measurement point toward the last, which is not guaranteed otherwise due to lingering artifacts. All fits are initiated from multiple randomized positions until a better fit could not be found for five consecutive attempts, evaluated based on root mean squared error (RMSE). Increasing this number over five did not yield significant improvements in the RMSE of the fitted curves.

The custom function that handles the fitting of Gaussian curves, including the “prepareCurveData” and “fit” functions, generates 3D tensors from which the Gaussian coefficients (amplitude, center, and standard deviation) and the “Goodness of Fit” metrics (root‐mean‐squared error, squares due to error, *R*‐square, degrees of freedom adjusted *R*‐square) are extracted for each selected order of fit from one to eight, and for each measurement point. For example, if the experimental data consist of 10 fluorescent profiles, and each of those profiles were fitted with the linear combination of eight Gaussian functions, there will be eight sets consisting of 10 Gaussian functions each, where each function belongs to a different measurement point, and their standard deviations, and thus their variances, form a linear, monotonically increasing function in time. Each of these monotonically increasing functions represent a particle size that is supposedly present within the measured analytes solution, and the diffusion coefficient associated with this particle size is one fourth of the incline of the associated monotonically increasing function. So, the final step the analytic software carries out before displaying the results is to fit a linear function to the monotonic function that displays the variances of the Gaussian function within the same set against the estimated average time particles spent between measurement points (Fig. 2). Then, the diffusion coefficients can be determined, and particle radii can be approximated (as described in the section Theory behind the applied diffusion analysis).

## Results

### Development of the microfluidic device

**Fig. 7:**
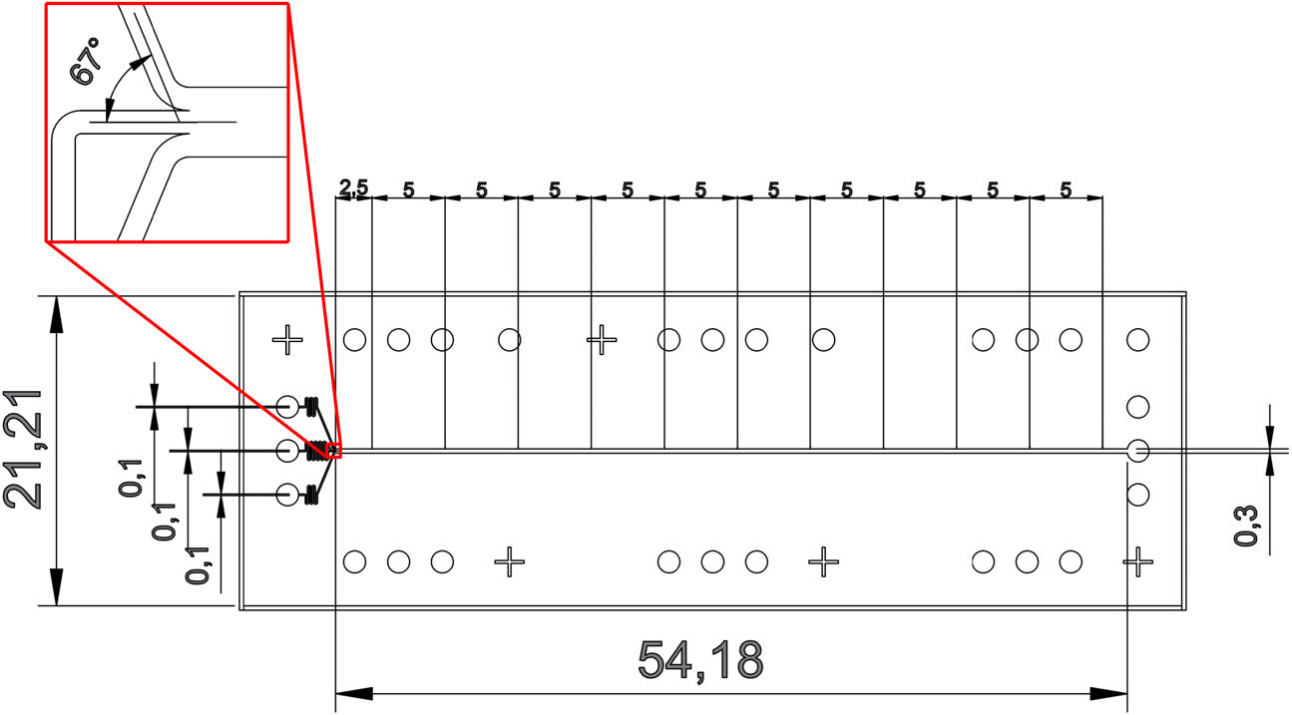
Layout of the microfluidic device that is three design units long, includes resistances on the inlets that meet at an angle of 67 °, and has a straight main channel that is wider than the inlets. There are eleven equidistant measurement points marked along the main channel. The channel height is 20 μm, the distances denoted in the image are in mm.

The final layout of the microfluidic device presented in Fig. 7 is the product of an iterative design process where devices of an initial design were manufactured, tested, and then modified based on observations. Previous layouts are included in the Data Accessibility section along with the detailed explanation of their shortcomings and gradual improvement. All layouts were designed in Autodesk AutoCAD.

The combination of a larger resistance on the middle inlet, smaller resistances on the side inlets, and an intersection with a 67 ° angle proved to minimize the probability of backflow, where the side streams would flow toward the middle inlet, halting the analyte that has to be injected at a lower flow rate. At the same time, this setup also maximized space for the main channel, and therefore particle diffusion. The main channel was kept straight to avoid the disruption of laminar flow, and it has been widened relative to the inlets to both further reduce the probability of backflow and to provide more space for lateral diffusion before particles reach its side walls. Through testing, it has been established that a 20× lens produces high enough quality images; therefore, devices could be bound to glass slides instead of the thinner cover plates that were compatible with the 60× lens. This allowed the elongation of the main channel by extending the device to three design units, the longest device that would fit onto a single glass slide. The main channel was also fitted with markers that assist in determining the exact distance between recorded intensity profiles, which show the lateral distribution of particles at different measurement points along the main channel.

### Size approximation

**Fig. 8:**
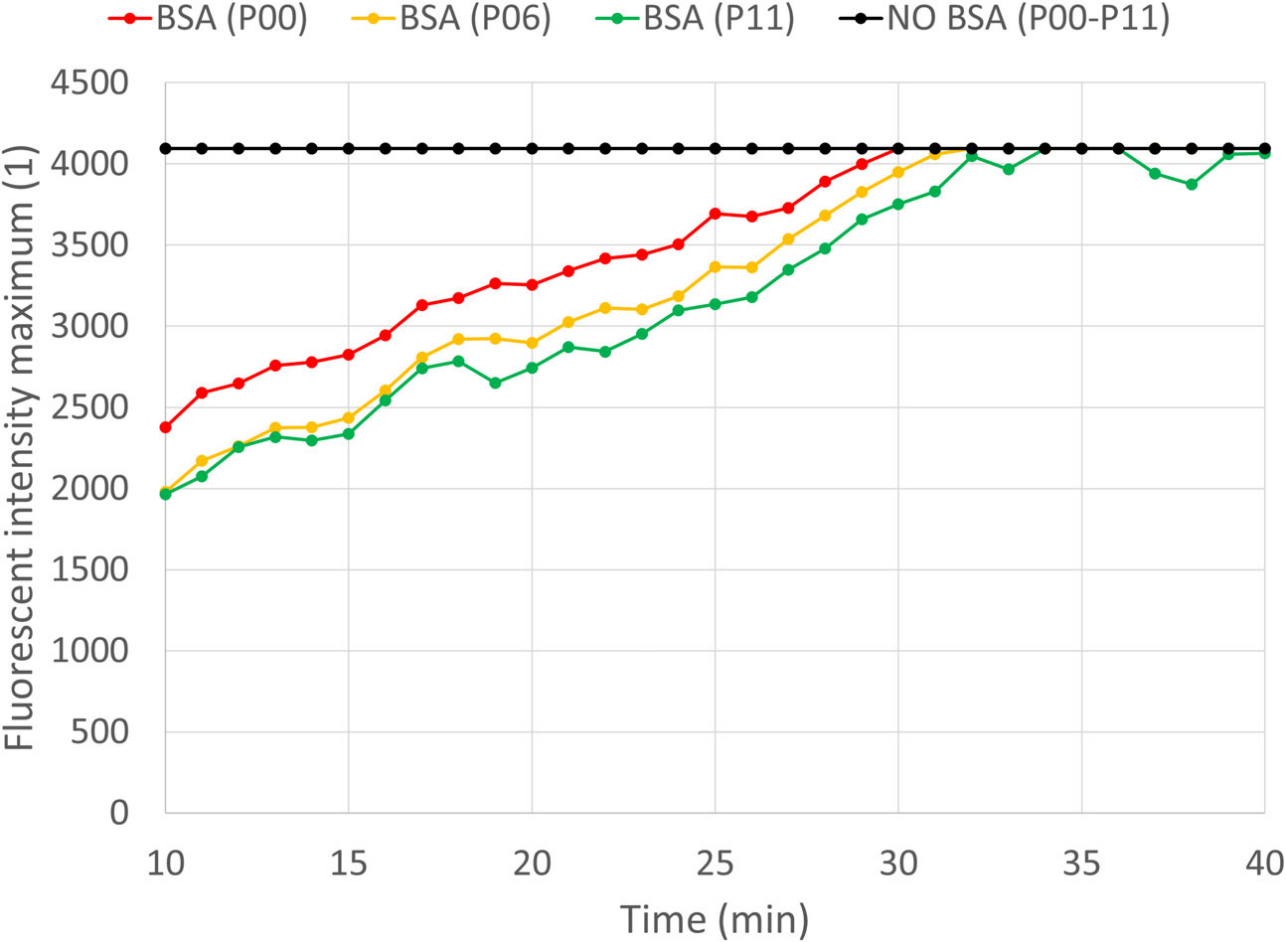
Effects of BSA (bovine serum albumin) treatment on particle accumulation on the surface of the device. Fluorescent intensity maxima were recorded at the front end of the main channel (P00), at its middle (P06), and at its rear end (P11), measured every minute for half an hour with and without treating the microfluidic device with BSA. In both cases, the first measurement took place 10 min after the analyte had reached the front end of the channel (see Measurement protocol). The analyte consisted of fluorescent microspheres with a nominal diameter of 0.05 μm; the measurements with and without BSA treatment were both conducted with a single biologically independent replicate (*n* = 1).

Based on observations, it takes 10–15 min for the microfluidic system to reach steady state after the analyte enters the device. Measurements can only be carried out after this waiting period, during which fluorescent particles accumulate on the surface of the channel. In the preliminary experiment shown in Fig. 8, peak fluorescent intensity is measured along the channel, 10–40 min after the analyte entered the device. The conclusion is that the fluorescence of the particles that accumulate all along the channel exceeds the limit of the microscope by the time measurements could take place, unless the surface of the device is treated with BSA, a practice incorporated in the measurement protocol (see Table 1).

**Fig. 9:**
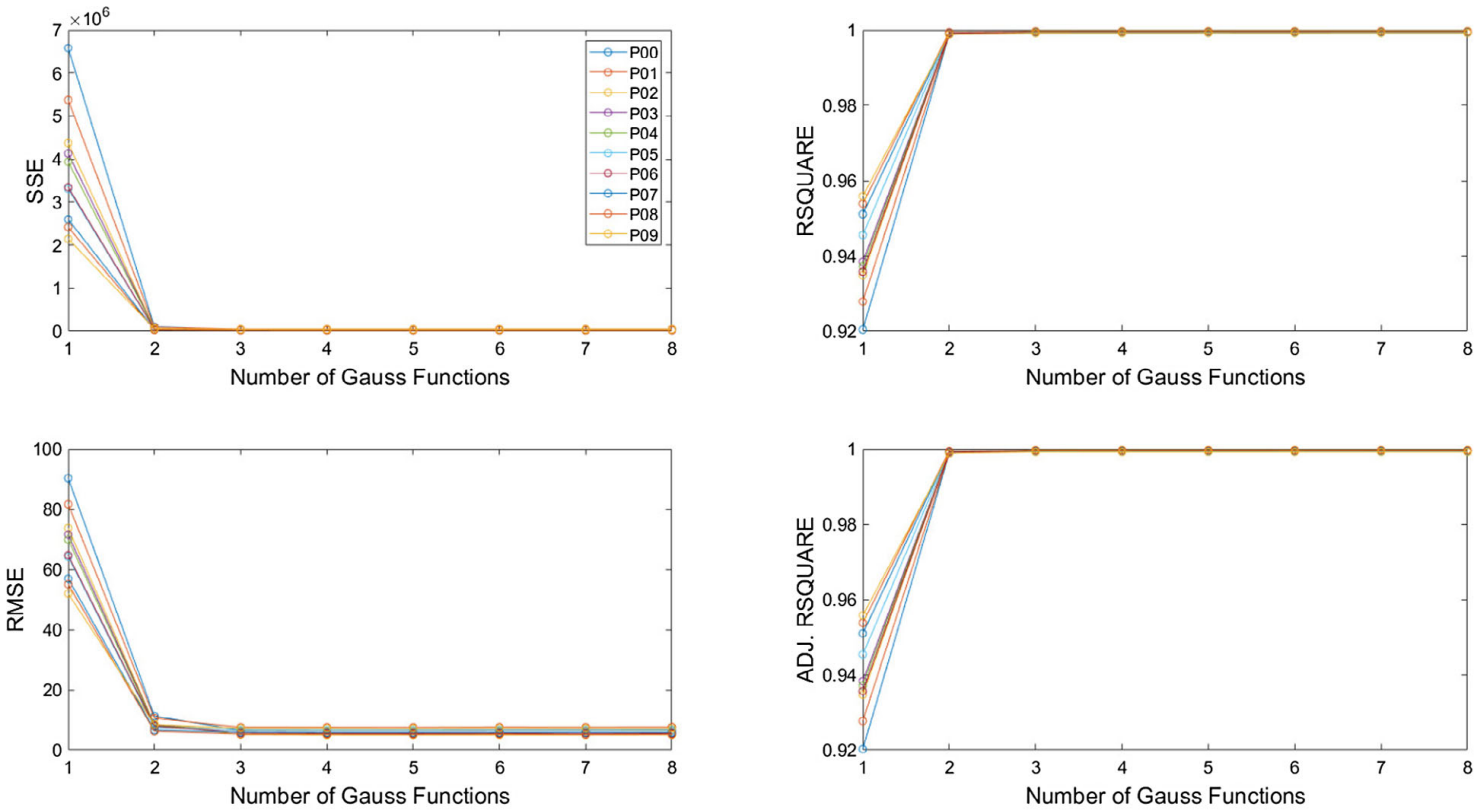
Goodness of fit (GoF) metrics, used to determine the best model for fitting Gaussian functions to measured fluorescent intensity profiles. Four GoF metrics were considered: sum of squares due to error (SSE), root mean squared error (RMSE), *R*‐square, and degrees of freedom adjusted *R*‐square. The models differed in the number of Gaussian functions that were combined to fit the measured data. The simplest model fitted a single Gaussian function to each profile, while the most complex model fitted the linear combination of eight Gaussian functions. The analyte was the enhanced green fluorescent protein for this particular measurement (*n* = 1), but all analytes (see Table 2.) exhibited a similar trend in all GoF metrics that proved consistent at different measurement points along the microfluidic device (P00–P09).

**Table 1:** Description of each syringe pump's settings during different phases of a measurement. Syringe pumps A and C are connected to the side inlets of the microfluidic device, while syringe pump B is connected to the middle inlet (corresponding to Fig. 3).

Measurement phase	Pump A		Pump B		Pump C	
	Solutions	Flow rate (μL·min^−1^)	Solutions	Flow rate (μL·min^−1^)	Solutions	Flow rate (μL·min^−1^)
Degassing with BSA	600 μL 1% BSA	20	600 μL 1% BSA	20	600 μL 1% BSA	20
BSA treatment		4		4		4
Degassing with buffer	400 μL PBS buffer	14		4	400 μL PBS buffer	14
Slowing buffer		4		4		4
Injecting analyte		4	300 μL analyte	4		4
Reaching steady state		4		1		4

**Table 2:** List of analytes, complete with their measured radii via DLS (dynamic light scattering) and the diffusion‐based approach. In the case of GKAP‐PBM (the guanylate kinase‐associated protein's PDZ‐binding motif) and D233 (Drebrin's 233–317 segment) the DLS measurements were replaced by approximations based on their molecular weights, respectively. Each set of DLS measurements yielded a mean hydrodynamic radius and an associated standard deviation. Each set is listed individually where applicable. The mean values and standard deviations for the diffusion‐based approach (approximate radii) were calculated from multiple measurements. EGFP (enhanced green fluorescent protein) samples were used for the calibration of the experimental setup. Postsynaptic density protein samples included D233, GKAP‐PBM, and GKAP‐DLC2 molecules (the guanylate kinase associated protein's dynein light chain LC8 protein‐binding motifs), as well as GKAP‐DLC2 + LC8 hexamers (consisting of two GKAP‐DLC2 molecules and two dynein light chain LC8 protein dimers), respectively. Drebrin and GKAP molecules were labeled with green‐fluorescent fluorescein‐EX (FITC). Since the analytic software was set to fit the linear combination of two Gaussian functions to the measured data (see Fig. 9), the output from each measurement was a pair of approximate radii. Column three includes both values, while columns four and five only include either the higher or the lower value of the same outpu.

Analyte	Expected radius (nm)	Approximate radius (nm)	Higher approx. radius (nm)	Lower approx. radius (nm)
EGFP	2.72 ± 1.01	1.72 ± 0.42	2.03 ± 0.20	1.42 ± 0.36
GKAP‐DLC2	1.27 ± 0.72 1.90 ± 1.00	1.36 ± 0.30	1.56 ± 0.24	1.15 ± 0.18
GKAP‐DLC2 + LC8	2.17 ± 0.44 3.11 ± 0.76	1.64 ± 0.36	1.91 ± 0.30	1.38 ± 0.27
GKAP‐PBM	1.39	1.16 ± 0.10	1.26 ± 0.03	1.06 ± 0.00
D233	1.81	1.45 ± 0.39	1.83 ± 0.09	1.06 ± 0.00

Four GoF metrics were considered when evaluating the different models: Sum of squares due to error (SSE), root mean squared error (RMSE), *R*‐square, and degrees of freedom adjusted *R*‐square. In the case of SSE and RMSE, a lower value corresponds to a better fit, while in the case of the other two metrics, the higher the value the better the fit. All four metrics showed that the model fitting a single Gaussian function is significantly worse than the one fitting the linear combination of two Gaussian functions. However, models that use more than two Gaussian functions did not result in significantly better fits. Since all analytes were assumed to be monodisperse, it was favorable to choose a simpler model. Therefore, measured profiles were fitted with the linear combination of two Gaussian functions, providing the quantified data used in the approximations below.

**Fig. 10:**
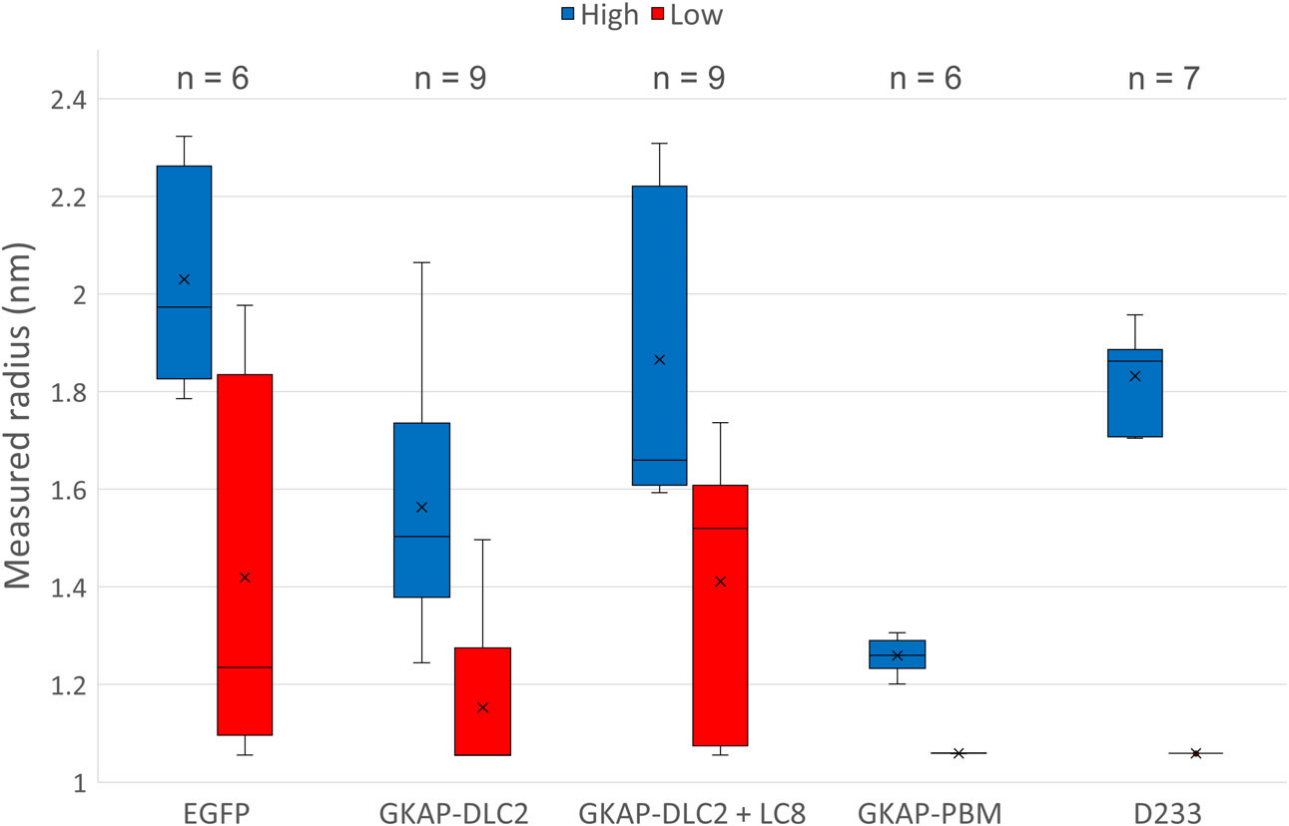
Box plot of measured radii for different particle types: EGFP (enhanced green fluorescent protein, *n* = 6), GKAP‐DLC2 (the guanylate kinase‐associated protein's two dynein light chain‐binding motifs, *n* = 9), GKAP‐DLC2 + LC8 (the hexameric complex formed by two GKAP‐DLC2 molecules and two dynein light chain LC8 protein dimers, *n* = 9), GKAP‐PBM (the guanylate kinase‐associated protein's PDZ‐binding domain, *n* = 6), D233 (Drebrin's 233–317 segment, *n* = 7). The number of independent measurements (*n*) is also shown above each analyte. Since the analytic software was set to fit the linear combination of two Gaussian functions to the measured data (see Fig. 9), the output from each measurement was a pair of approximate radii, the minimum boundary for which was set to 1 nm in the analytic software. Due to rounding errors in the various operations executed on the measured data, the actual lower limit is about 1.05 nm (see The analytic software package). This is reflected by the boxes that contain the lower value from each pair of radii (red). The boxes that contain the higher value from each pair of radii (blue) adhere much better to the values yielded by the control measurements (see Table 2). The boxes themselves represent the interquartile range (IQR), calculated with exclusive median. The whiskers extend to the furthest datapoints that are within 1.5*IQR of the quartiles.

The approximate radii are quite close to their expected values for such small particles. However, since the diffusion of smaller particles is much more significant at the same flow rate, the method might be biased toward them when fitting Gaussian functions to the measured profiles. This is somewhat mitigated by discarding the lower value from the output (see Fig. 10). Both DLS and the diffusion‐based approach reflect that GKAP‐DLC2 + LC8 hexamers are larger than GKAP‐DLC2 monomers, which are in turn larger than GKAP‐PBM domains. However, in the case of DLS, it is unclear whether EGFP molecules are larger than GKAP‐DLC2 + LC8 hexamers. In all cases, the standard deviation is lower for the diffusion‐based approach, highlighting that at the ends of the 1 nm to 10 μm range of DLS (Zetasizer Ultra/Pro), its precision becomes increasingly system‐dependent.

Fig. 10 highlights the bias of this diffusion‐based technique. While the higher approximations correspond well with the control measurements carried out via DLS, the lower values from each output are skewed toward the preset lower limit of 1 nm. The standard deviation of the lower values seems to be loosely proportional to that of the higher radii for each type of analyte particle. The standard deviation also exhibits a trend of increasing with particle size. This is to be expected since larger particles diffuse at a lower rate, and so their distribution changes less significantly along the main channel of the microfluidic device.

## Discussion

We have developed a diffusion‐based approach for the measurement of solute particle sizes, combining microfluidics and fluorescent microscopy techniques. The main difference between our approach and similar methods published by Arosio *et al*. and Gang *et al*. [28, 29] is that our data analysis pipeline does not require *a priori* knowledge of the particles within the solution. Our approach is in principle suitable for the investigation of proteins and their complexes that, via polymerization and multivalent interactions, occupy a dynamic range of particle sizes. For this purpose, we have developed a novel microfluidic device and analytic software that converts measured data into approximate particle radii. Our approach proved to be a working concept that has some limitations in the precise distinction between particles of closely similar sizes.

The method is biased toward smaller particles present in the solution, and the results yielded by it must be cross‐referenced with those of samples with known particle sizes. The method provides accurate approximations for particles below 10 nm in diameter, a size range where even established techniques such as DLS may struggle. It becomes increasingly inaccurate for larger particles, yielding completely wrong approximations for particles in the 1 μm range and above. Notably, it yielded accurate results for solutions with unintended polydispersity, arising from the polymerization of multivalent proteins with disordered segments. However, the limited range of particle sizes that can be measured this way is a definitive disadvantage.

The length of the PTFE tubing connected to the microfluidic device's inlets could be further decreased with the fabrication of specialized equipment that holds the syringe pumps closer to the device and mimics the motions of the stage without interfering with the microscope. This would reduce the time it takes for the solutions to reach the device as well as the minimum volume of solutions required for a measurement.

Ultimately, the method in its current state cannot be expected to reliably distinguish the observed proteins from their complexes in each case, but it is sufficient for distinguishing proteins and their complexes from larger particles and provides a size approximation that is well in the range determined by other methods. The accurate measurement of larger particles would require a more refined model that describes signal forms with polynomial functions, creating a library from particles of known sizes to which new data could be compared. This improved method would still be limited, however, to the measurement of particles that conform to already acquired data that can only cover a set of distinct sizes.

In the case of PSD proteins and complexes, the method is in principle capable of assessing the overall size of possible assemblies. For the GKAP:LC8 complex, the results are in line with our previous observations that this system has a well‐defined stoichiometry and the presence of substantial heterogeneity in terms of assembly size can largely be excluded. On the other hand, the large standard deviation in the measurements is likely attributable, at least in part, to the flexibility of the complex that might influence its diffusion properties under the conditions applied. Notably, the estimated radii for the two shorter disordered segments are in very good agreement with the expectations. Further optimization of the experimental setup and improved prior estimates of the diffusion properties of the particles are expected to render the method applicable for a wide range of multivalent and dynamic protein complexes.

## Conflict of interest

The authors declare no conflict of interest.

## Author contributions

Z.G. and A.L.S. designed the study; M.L., A.J.L., and A.L.S. developed the device; E.N.K., S.V., and E.A.J. prepared the samples; A.L.S., E.A.J., and C.I.P. carried out the measurements; A.L.S. developed the analytic software; Z.G., A.L.S., and E.N.K. analyzed the data; all authors contributed to data interpretation, drafting the article, and approved the final version.

## Data Availability

Measured fluorescent and bright‐field intensity profiles that support the findings of this study are available in the following Zenodo dataset: doi:10.5281/zenodo.15394328. The analytic software created to process the measured intensity profiles is available in the following Zenodo software package: doi:10.5281/zenodo.15394359. Previous layouts of the microfluidic device, along with the description of its gradual development, are available as the following collection of figures: doi:10.5281/zenodo.15773654.
